# HIV Testing, Linkage to HIV Medical Care, and Interviews for Partner Services Among Women — 61 Health Department Jurisdictions, United States, Puerto Rico, and the U.S. Virgin Islands, 2015

**DOI:** 10.15585/mmwr.mm6641a2

**Published:** 2017-10-20

**Authors:** Renee Stein, Songli Xu, Mariette Marano, Weston Williams, Qi Cheng, Adanze Eke, Andrea Moore, Guoshen Wang

**Affiliations:** 1 Division of HIV/AIDS Prevention, National Center for HIV/AIDS, Viral Hepatitis, STD, and TB Prevention, CDC.

Diagnoses of human immunodeficiency virus (HIV) infection among women declined 17% during 2011–2015, and a total of 7,498 women received a diagnosis of HIV infection in 2015 (*1*). Although black or African American (black) women accounted for only 12% of the U.S. female population, 60% of women with newly diagnosed HIV infection were black (*1*,*2*). By the end of 2014, an estimated 255,900 women were living with HIV infection (*3*), including approximately 12% who did not know they were infected; in addition, approximately 45% of women who had received a diagnosis had not achieved viral suppression (*3*). HIV testing is an important public health strategy for identifying women with HIV infection and linking them to HIV medical care. Analysis of CDC-funded program data submitted by 61 health departments in 2015 indicated that among 4,749 women tested who received a diagnosis of HIV infection, 2,951 (62%) had received a diagnosis in the past (previous diagnosis), and 1,798 (38%) were receiving a diagnosis for the first time (new diagnosis). Of those who had received a previous diagnosis, 87% were not in HIV medical care at the time of the current test. Testing and identifying women who are living with HIV infection but who are not in care (regardless of when they received their first diagnosis) and rapidly linking them to care so they can receive antiretroviral therapy and become virally suppressed are essential for reducing HIV infection among all women.

In 2015, CDC funded 61 state and local health departments and 123 community-based organizations (CBOs)* to provide HIV testing and related services in the United States, Puerto Rico, and the U.S. Virgin Islands. Health departments submitted deidentified program data about services provided by both health departments and CBOs through a secure, online, CDC-supported system. Data analyzed for this report include 2015 CDC-funded HIV tests,^†^ new and previous HIV diagnoses, linkage to medical care within 90 days^§^ of the current test, and interviews for partner services.^¶^ Analyses were restricted to persons who reported their sex at birth and current gender identity as female, and were aged ≥13 years. Data were stratified by the following demographic characteristics: age group, race/ethnicity, census region, health department jurisdiction’s prevalence of HIV infection, and test setting.** Multivariate robust Poisson regression (*4*) was used to assess the association between demographic characteristics and newly diagnosed HIV infections, linkage to HIV medical care, and interviews for partner services.

Among 3,020,068 CDC-funded HIV tests provided in 2015, a total of 1,454,499 (48%) were provided to women. The highest percentages of tests were provided to women who were aged 20–29 years (41%), black (49%), living in the South (62%), living in medium and high prevalence jurisdictions (97%), and who received testing in health care facilities (83%) (Table 1).

**TABLE 1:** HIV tests and newly diagnosed HIV infections among women by selected characteristics — United States, Puerto Rico, and the U.S. Virgin Islands, 2015

Characteristic	HIV tests,* no. (% of total [column %])	Newly diagnosed HIV infections^†^			
		No.	(% of total [column %])	(% of category [row %])	aPR (95% CI)
**Age group (yrs)**					
13–19	131,547 (9.08)	43	(2.40)	(0.03)	0.41 (0.30–0.57)^¶^
20–29	599,777 (41.38)	493	(27.47)	(0.08)	Referent
30–39	347,016 (23.94)	472	(26.30)	(0.14)	1.69 (1.49–1.93)^¶^
40–49	185,523 (12.80)	392	(21.84)	(0.21)	2.58 (2.25–2.95)^¶^
≥50	185,612 (12.81)	395	(22.01)	(0.21)	2.53 (2.22–2.90)^¶^
**Race/Ethnicity**					
White	339,714 (24.82)	393	(22.39)	(0.12)	Referent
Black	666,322 (48.68)	1,018	(58.01)	(0.15)	1.31 (1.17–1.48)^¶^
Hispanic	318,456 (23.26)	291	(16.58)	(0.09)	0.65 (0.55–0.75)^¶^
Asian	25,941 (1.90)	17	(0.97)	(0.07)	0.46 (0.28–0.75)^§^
American Indian	7,086 (0.52)	15	(0.85)	(0.21)	1.41 (0.82–2.43)
Native Hawaiian	2,439 (0.18)	7	(0.40)	(0.29)	1.96 (0.93–4.13)
Multiple races	8,872 (0.65)	14	(0.80)	(0.16)	1.28 (0.75–2.19)
**U.S. Census region**					
Northeast	210,472 (14.47)	349	(19.41)	(0.17)	1.65 (1.46–1.88)^¶^
Midwest	194,856 (13.40)	243	(13.52)	(0.12)	1.24 (1.08–1.43)^§^
South	895,271 (61.55)	956	(53.17)	(0.11)	Referent
West	129,530 (8.91)	217	(12.07)	(0.17)	1.69 (1.45–1.98)^¶^
Puerto Rico and U.S. Virgin Islands	24,370 (1.68)	33	(1.84)	(0.14)	2.26 (1.56–3.27)^¶^
**HIV prevalence**					
High	833,892 (57.33)	1,149	(63.90)	(0.14)	Referent
Medium	580,710 (39.93)	627	(34.87)	(0.11)	0.86 (0.78–0.95)^§^
Medium-low/Low	39,897 (2.74)	22	(1.22)	(0.06)	0.38 (0.24–0.58)^¶^
**Test setting**					
Health care facility	1,206,078 (83.11)	1,262	(70.46)	(0.10)	0.51 (0.46–0.57)^¶^
Non–health care facility	245,109 (16.89)	529	(29.54)	(0.22)	Referent
**Total**	**1,454,499 (100.00)**	**1,798**	**(100.00)**	**(0.12)**	**—**

**Abbreviations:** aPR = adjusted prevalence ratio; CI = confidence interval; HIV = human immunodeficiency virus.

* Valid HIV tests were defined as tests for which a test result (i.e., positive or negative) was known. Analyses excluded discordant and indeterminate results. When data are stratified by age group, race/ethnicity, and test setting; missing or invalid values are not shown in the table.

^†^ Included persons who tested HIV-positive during the current test and were not previously reported in the health department jurisdiction’s HIV surveillance system or who self-reported not having a previous HIV-positive test result if surveillance system verification was not available.

^§^ p<0.01.

^¶^ p<0.001.

Overall, 4,749 women had positive tests for HIV infection in 2015; among these, 2,951 (62%) had previously received a diagnosis of HIV infection, and 1,798 (38%) received a new diagnosis. Compared with women aged 20–29 years, those aged 13–19 years were less likely to receive a new diagnosis (adjusted prevalence ratio [aPR] = 0.41), whereas prevalence was higher among women aged 30–39 years (aPR = 1.69), 40–49 years (2.58), and ≥50 years (2.53). Black women accounted for 58% of the new diagnoses of HIV infection and were more likely to receive a new diagnosis than were white women (aPR = 1.31). Compared with tests performed in the South, tests performed in in the Northeast, Midwest, West, and Puerto Rico and the U.S. Virgin Islands were more likely to yield new diagnoses (aPR = 1.65, 1.24, 1.59, and 2.26, respectively). Compared with tests performed in high prevalence jurisdictions, tests performed in medium and medium-low/low prevalence jurisdictions were less likely to yield new diagnoses (aPR = 0.86 and 0.38, respectively). Tests performed in health care facilities were less likely to yield new diagnoses than tests performed in non–health care facilities (aPR = 0.51) (Table 1).

Among the 1,798 women with newly diagnosed HIV infection, 1,104 (61%) were linked to HIV medical care within 90 days of diagnosis, and 1,096 (61%) were interviewed for partner services. The percentages of women with newly diagnosed HIV infection who were linked to care were higher in the Northeast (72%; aPR = 1.21) and Puerto Rico and the U.S. Virgin Islands (79%; aPR = 1.57) than in the South (59%) (Table 2).

**TABLE 2:** Linkage to HIV medical care and interview for partner services among women with newly diagnosed HIV infection, by selected characteristics — United States, Puerto Rico, and the U.S. Virgin Islands, 2015

Characteristic	No. newly diagnosed HIV infections*	Linked to HIV medical care within 90 days of diagnosis^†^			Interviewed for partners services^§^		
		No. (%)	aPR (95% CI)	No. missing linkage information (%)	No. (%)	aPR (95% CI)	No. missing linkage information (%)
**Age group (yrs)**							
13–19	43	27 (62.79)	1.00 (0.80–1.27)	12 (27.91)	24 (55.81)	0.92 (0.70–1.21)	8 (18.60)
20–29	493	306 (62.07)	Referent	133 (26.98)	296 (60.04)	Referent	83 (16.84)
30–39	472	290 (61.44)	0.97 (0.88–1.07)	115 (24.36)	289 (61.23)	1.02 (0.92–1.13)	98 (20.76)
40–49	392	242 (61.73)	0.96 (0.86–1.06)	111 (28.32)	256 (65.31)	1.08 (0.97–1.20)	72 (18.37)
≥50	395	237 (60.00)	0.89 (0.80–0.99)^¶^	116 (29.37)	229 (57.97)	0.94 (0.84–1.05)	89 (22.53)
**Race/Ethnicity**							
White	393	225 (57.25)	Referent	114 (29.01)	236 (60.05)	Referent	67 (17.05)
Black	1,018	642 (63.06)	1.05 (0.95–1.16)	256 (25.15)	623 (61.20)	1.01 (0.92–1.12)	201 (19.74)
Hispanic	291	189 (64.95)	1.00 (0.87–1.13)	77 (26.46)	193 (66.32)	1.01 (0.89–1.15)	51 (17.53)
Asian	17	10 (58.82)	0.97 (0.64–1.47)	6 (35.29)	9 (52.94)	0.85 (0.54–1.34)	6 (35.29)
American Indian	15	10 (66.67)	1.09 (0.77–1.55)	3 (20.00)	9 (60.00)	1.07 (0.73–1.56)	2 (13.33)
Native Hawaiian	7	4 (57.14)	0.88 (0.46–1.69)	2 (28.57)	3 (42.86)	0.62 (0.26–1.49)	1 (14.29)
Multiple races	14	9 (64.29)	1.16 (0.81–1.67)	5 (35.71)	6 (42.86)	0.73 (0.41–1.33)	4 (28.57)
**U.S. Census region**							
Northeast	349	252 (72.21)	1.21 (1.11–1.32)^††^	56 (16.05)	217 (62.18)	1.03 (0.93–1.13)	90 (25.79)
Midwest	243	127 (52.26)	0.99 (0.86–1.14)	74 (30.45)	139 (57.20)	1.04 (0.90–1.19)	37 (15.23)
South	956	562 (58.79)	Referent	286 (29.92)	568 (59.41)	Referent	199 (20.82)
West	217	137 (63.13)	1.09 (0.96–1.23)	66 (30.41)	147 (67.74)	1.12 (1.00–1.25)	17 (7.83)
Puerto Rico and U.S. Virgin Islands	33	26 (78.79)	1.57 (1.26–1.95)^††^	6 (18.18)	25 (75.76)	1.39 (1.09–1.78)**	7 (21.21)
**HIV prevalence**							
High	1,149	748 (65.10)	Referent	287 (24.98)	732 (63.71)	Referent	208 (18.10)
Medium	627	336 (53.59)	0.81 (0.74–0.89)^††^	200 (31.90)	345 (55.02)	0.84 (0.76–0.93) ^††^	141 (22.49)
Medium–low/Low	22	20 (90.91)	1.42 (1.19–1.69)^††^	1 (4.55)	19 (86.36)	1.31 (1.06–1.61) ^¶^	1 (4.55)
**Test setting**							
Health care facility	1,262	791 (62.68)	1.05 (0.96–1.14)	348 (27.58)	776 (61.49)	1.03 (0.95–1.13)	256 (20.29)
Non–health care facility	529	311 (58.79)	Referent	137 (25.90)	316 (59.74)	Referent	94 (17.77)
**Total**	**1,798**	**1,104 (61.40)**	**—**	**488 (27.14)**	**1,096 (60.96)**	**—**	**350 (19.47)**

**Abbreviations**: aPR = adjusted prevalence ratio; CI = confidence interval; HIV = human immunodeficiency virus.

*Included persons who tested HIV-positive during the current test and were not found to be previously reported in the health department jurisdiction’s HIV surveillance system or self-reported not having a previous HIV-positive test result if surveillance system verification is not available. When data are stratified by age group, race/ethnicity, and test setting; missing or invalid values are not shown in the table.

^†^ Linkage to HIV medical care within 90 days of diagnosis means confirmation that the person attended their first HIV medical care appointment within 90 days of their HIV test date.

^§^ Partner services is a process through which HIV infected persons are interviewed to elicit information about their partners, who can then be confidentially notified of their possible exposure or potential risk and offered services that can protect the health of partners and prevent HIV transmission to others.

^¶^ p<0.05.

** p<0.01.

^††^ p<0.001.

Among the 2,951 women with a previously diagnosed infection, 2,554 (87%) were not in HIV medical care at the time of testing (Table 3); among these women, 1,474 (58%) were linked to care within 90 days. The prevalence of being linked to HIV medical care within 90 days was lower for black women (57%) than for white women (65%; aPR = 0.91). The prevalence of being linked to care was 53% in the South, and was higher in the Northeast (78%; aPR = 1.46) and the West (86%; aPR = 1.57). Compared with women tested in non–health care facilities (68%), linkage was lower among women tested in health care facilities (55%; aPR = 0.80) (Table 3).

**TABLE 3:** Linkage to HIV medical care among women with previously diagnosed HIV infection, by selected characteristics — United States, Puerto Rico, and the U.S. Virgin Islands, 2015

Characteristic	Previously diagnosed HIV infections*	Not in HIV medical care at time of HIV test	Women with previously diagnosed HIV infection (not in HIV medical care at time of test) linked to HIV medical care^†^		
	No.	No. (%)	No. (%)	aPR (95% CI)	Missing linkage info. No. (%)
**Age group (yrs)**					
13–19	38	35 (92.11)	26 (74.29)	1.20 (0.97–1.48)	2 (5.71)
20–29	564	520 (92.20)	307 (59.04)	Referent	154 (29.62)
30–39	796	700 (87.94)	422 (60.29)	0.99 (0.90–1.09)	184 (26.29)
40–49	740	631 (85.27)	357 (56.58)	0.94 (0.85–1.04)	200 (31.70)
≥50	811	666 (82.12)	362 (54.35)	0.92 (0.84–1.02)	212 (31.83)
**Race/Ethnicity**					
White	400	365 (91.25)	236 (64.66)	Referent	84 (23.01)
Black	2,047	1,730 (84.51)	978 (56.53)	0.91 (0.84–0.99)^§^	536 (30.98)
Hispanic	350	308 (88.00)	201 (65.26)	0.98 (0.87–1.10)	80 (25.97)
Asian	18	18 (100.00)	13 (72.22)	1.09 (0.83–1.44)	5 (27.78)
American Indian	10	10 (100.00)	5 (50.00)	0.74 (0.41–1.34)	2 (20.00)
Native Hawaiian	5	5 (100.00)	4 (80.00)	1.06 (0.71–1.57)	N/A
Multiple races	10	9 (90.00)	6 (66.67)	1.07 (0.64–1.80)	3 (33.33)
**U.S. Census region**					
Northeast	277	254 (91.70)	197 (77.56)	1.46 (1.34–1.59)^¶^	35 (13.78)
Midwest	226	191 (84.51)	110 (57.59)	1.07 (0.93–1.21)	49 (25.65)
South	2,281	1,959 (85.88)	1,044 (53.29)	Referent	650 (33.18)
West	135	125 (92.59)	107 (85.60)	1.57 (1.43–1.73)^¶^	13 (10.40)
Puerto Rico and U.S. Virgin Islands	32	25 (78.13)	16 (64.00)	1.12 (0.83–1.53)	7 (28.00)
**HIV prevalence**					
High	1,804	1,620 (89.80)	946 (58.40)	Referent	477 (29.44)
Medium	1,130	920 (81.42)	518 (56.30)	1.09 (1.01–1.18)^§^	274 (29.78)
Medium-low/Low	17	14 (82.35)	10 (71.43)	1.11 (0.79–1.56)	3 (21.43)
**Test setting**					
Health care facility	2,350	1,996 (84.94)	1,095 (54.86)	0.80 (0.75–0.86)^¶^	653 (32.72)
Non–health care facility	598	555 (92.81)	378 (68.11)	Referent	99 (17.84)
**Total**	**2,951**	**2,554 (86.55)**	**1,474 (57.71)**	**—**	**754 (29.52)**

**Abbreviations:** aPR = adjusted prevalence ratio; CI = confidence interval; HIV = human immunodeficiency virus.

* Previously diagnosed HIV infections included those in women who tested HIV-positive during the current test and were found to have been previously reported in the health department’s HIV surveillance system or, if the surveillance system verification is not available, self-reported having a previous HIV-positive test result. When data are stratified by age group, race/ethnicity, and test setting; missing or invalid values are not shown in the table.

^†^ Linkage to HIV medical care within 90 days of diagnosis means confirmation that the person attended their first HIV medical care appointment within 90 days of their HIV test date.

^§^ p<0.05.

^¶^ p<0.001.

## Discussion

HIV testing and partner services are essential strategies for diagnosing and rapidly linking women living with HIV infection and their infected partners to medical care so they can achieve viral suppression. Findings from this report underscore the importance of HIV testing not only to identify new infections, but also to identify women who have previously received a diagnosis but are not receiving medical care, because either they were never linked to care or they stopped receiving care. A high percentage of women tested had already received an HIV diagnosis (62%) before the current test; however, 87% of those women were not receiving HIV medical care. Willingness of women with previously diagnosed HIV infection to take another HIV test might signal a desire or willingness to receive the additional support they need to be linked to care, representing an opportunity for an important public health intervention; however, in this analysis, only 58% of women with previously diagnosed HIV infection were linked to care within 90 days of the current test.

Black women accounted for 72% of women with a previous HIV diagnosis, although they were significantly less likely than were white women to be linked to HIV medical care within 90 days of the current test. Disparities in socioecological factors such as poverty, health literacy, and health care coverage might contribute to lower linkage for black women (*5*). Mistrust of medical providers and conspiracy beliefs about the origin of HIV and the role of the government in the acquired immunodeficiency syndrome epidemic might also explain why black women are less likely to be engaged in HIV medical care (*6*). Black women might be more likely to remain in HIV medical care if they trust and engage in high quality communication with their provider (*7*).

Overall, 61% of women with newly diagnosed HIV infection were interviewed for partner services. CDC recommends that all persons with newly diagnosed HIV infection be interviewed for partner services so that partners can be confidentially notified of their potential risk (*8*). There are potential prevention benefits of interviewing women with previously diagnosed infection for partner services as well so that their partners may also be confidentially notified of their potential risk, receive an HIV test, and be linked to care if they receive a diagnosis of HIV. It is important to prioritize provision of partner services to women whose characteristics suggest possible recent risk for transmission (e.g., new bacterial sexually transmitted infections, pregnancy, report of sex without condoms, or sharing drug injection equipment) or those who did not receive partner services when they initially received their diagnosis (*8*). Partner services is a process through which HIV infected persons are interviewed to elicit information about their partners, who can then be confidentially notified of their possible exposure or potential risk and offered services that can protect the health of partners and prevent HIV transmission to others.

The findings in this report are subject to at least three limitations. First, findings describe CDC-funded HIV tests only and are not generalizable to all tests provided to women in the United States. Second, linkage data include records with missing or invalid data in the denominator and therefore probably underestimate the percentage of persons linked to care. Finally, when surveillance data are unavailable to verify previous HIV status, the number of new positive results might be overestimated if clients inaccurately report a previous negative HIV status.

To reduce and eventually eliminate HIV infection among women in the United States, HIV testing programs need to improve early linkage to HIV medical care among HIV-positive women who are not in care, regardless of their known HIV status at the time of testing. It is also important for the HIV prevention public health community to increase their focus on identifying women with previously diagnosed HIV infection who are not in care, especially black women, and promptly link them to care, as well as monitor and evaluate these efforts.

**Summary**

**What is already known about this topic?**

In 2015, a total of 7,498 women received a diagnosis of human immunodeficiency virus (HIV) infection in the United States, 60% of whom were black, although black women accounted for only 12% of the female population. HIV testing, identification of HIV infections, and early linkage to HIV medical care are critical for ensuring that HIV-positive women receive the care they need to achieve viral suppression and improved health outcomes, and to reduce transmission to others. Providing partner services can further support these prevention goals.

**What is added by this report?**

Analysis of 2015 data on CDC-funded HIV tests and HIV prevention services from 61 health departments and 123 community-based organizations indicated that among women identified as having HIV infection, 62% had received a diagnosis of HIV infection before the current test, and 87% of those women were not in HIV medical care at the time of the test. Rates for linkage to medical care within 90 days of the current test date were 61% and 58% for women with newly diagnosed and previously diagnosed HIV infection, respectively. Among women with previously diagnosed HIV infection, 57% of black women and 65% of white women were linked to HIV medical care.

**What are the implications for public health practice?**

Enhanced efforts to test and identify women with HIV infection and promptly link them to HIV medical care, as well as to identify women with previously diagnosed HIV infection who are not in care, especially black women, and link them to care will improve health outcomes, increase rates of viral suppression, and reduce transmission of HIV to others.

## Footnotes

* CDC-funded partners include health departments in the 50 states, the District of Columbia, Puerto Rico, the U.S. Virgin Islands, and eight directly funded city/county health departments (Baltimore, Maryland; Chicago, Illinois; Fulton County, Georgia; Houston, Texas; Los Angeles County, California; New York City, New York; Philadelphia, Pennsylvania; and San Francisco, California) and 123 directly funded community-based organizations. Community-based organizations report their National HIV Prevention Program Monitoring and Evaluation HIV testing data to their jurisdiction’s health department, who then submit them to CDC.

† An human immunodeficiency (HIV) test is defined as the performance of one or more HIV tests to determine a person’s HIV infection status. A person might be tested once (e.g., one rapid test or one conventional test) or multiple times (e.g., one rapid test followed by one conventional test to confirm a preliminary HIV-positive test result).

§ Linkage to HIV medical care within 90 days of diagnosis means confirmation that the person attended their first HIV medical care appointment within 90 days of their HIV test date.

¶ Partner services is a process through which HIV infected persons are interviewed to elicit information about their partners, who can then be confidentially notified of their possible exposure or potential risk and offered services that can protect the health of partners and prevent HIV transmission to others.

** Jurisdictions are grouped by HIV prevalence as determined by the number of persons living with diagnosed HIV infection in 2013, as follows: high: ≥20,000 persons living with HIV infection, medium: 4,000–19,999 persons living with HIV infection, medium–low/low: <3,999 persons living with HIV infection. High prevalence jurisdictions include California, Los Angeles, San Francisco, Florida, Georgia, Fulton County (Atlanta), Illinois, Chicago, Maryland, Baltimore, New Jersey, New York, New York City, North Carolina, Pennsylvania, Philadelphia, Texas, Houston, and Virginia. Medium prevalence jurisdictions include Alabama, Arizona, Arkansas, Colorado, Connecticut, District of Columbia, Indiana, Kentucky, Louisiana, Massachusetts, Michigan, Minnesota, Mississippi, Missouri, Nevada, Ohio, Oklahoma, Oregon, Puerto Rico, South Carolina, Tennessee, Washington, and Wisconsin. Medium–low/low prevalence jurisdictions include Alaska, Delaware, Hawaii, Idaho, Iowa, Kansas, Maine, Montana, Nebraska, New Hampshire, New Mexico, Rhode Island, South Dakota, U.S. Virgin Islands, Utah, Vermont, West Virginia, and Wyoming.
